# All-dielectric metaoptics for the compact generation of double-ring perfect vector beams

**DOI:** 10.1515/nanoph-2023-0555

**Published:** 2023-10-30

**Authors:** Andrea Vogliardi, Gianluca Ruffato, Daniele Bonaldo, Simone Dal Zilio, Filippo Romanato

**Affiliations:** Department of Physics and Astronomy ‘G. Galilei’, University of Padova, via Marzolo 8, 35131, Padova, Italy; Department of Information Engineering, University of Padova, via Gradenigo 6, 35131, Padova, Italy; CNR-IOM Istituto Officina dei Materiali, S.S. 14 – Km. 163, 5 – 34149, Trieste, Italy

**Keywords:** metasurfaces, orbital angular momentum, vector beams, perfect vortices, perfect vector beams, double-ring perfect vortices

## Abstract

Perfect vortices, whose ring profile is independent of the topological charge, play a key role in telecommunications and particle micro-manipulation. In this work, we report the compact generation of a new kind of double-ring perfect vortices, called double-ring perfect vector beams, by exploiting dual-functional silicon metaoptics. In particular, we develop and test a new paradigm to generate those beams with the possibility of selecting different topological charges between the two rings. The generated beams are characterized through a filtering method, proving that the two rings have a vectorial nature with the same magnitude and either the same or different topological charges. Their unique properties suggest promising applications for optical tweezing and manipulation of low refractive-index particles, trapping of cold atoms, and high-capacity communications.

## 1 Introduction

Since Coullet et al. proposed the concept of optical vortices in 1989 [1], the field of structured light [2] has routed a new understanding of a wide range of optical phenomena and inspired tremendous advanced applications in many areas, while the associated technologies for light manipulation witnessed increasing levels of versatility and integration [3]. Typical vortex beams such as Bessel [4], Kummer [5], and Laguerre–Gaussian beams [6] are endowed with axially-symmetric intensity profiles and intertwined helical wavefronts. Those unique properties routed disruptive applications in many fields, such as high-resolution microscopy [7], particle tweezing [8] and cold-atoms manipulation [9], high capacity optical communications [10], computer-generated holograms [11], and quantum encryption [12].

However, the ring parameters of those beams strongly depend on the carried topological charge, limiting their full exploitation in crucial scenarios. For instance, that may result in a low coupling efficiency when the vortex beam illuminates a ring-core fiber for high-order mode excitation [13]. Moreover, optical trapping requires fixed paths and high values of topological charge on small ring diameters [14]. In order to overcome those issues, Ostrovsky et al. [15] firstly proposed the generation of perfect vortices (PVs) with OAM-independent ring intensity patterns, obtained via the Fourier transform of a Gaussian beam illuminating an axicon and a spiral phase plate in cascade [16]. Concurrently, the non-separable combination of orbital and spin angular momenta in the so-called vector beams [17] introduced complex beams carrying spatially-variant polarization patterns with intriguing properties for imaging [18], lithography [19], rotation-invariant [20] and high-dimensional quantum communications [21, 22]. The same paradigm extended to perfect vortices, leading to the generation of perfect vector beams (PVBs), which exhibit an azimuthally-rotating polarization axis over a customizable intensity ring [23].

Nevertheless, previous investigations were only focused on perfect vortices having a single bright ring, which prevents the manipulation of low-refractive index (RI) particles [24], or the multiple trapping on different paths with different rotational speeds [25]. A potential solution is provided by double-ring perfect vortices (DR-PVs), where the two bright rings can trap and rotate the high-RI particles, while low-RI particles can be confined in the dark region in between. In addition, dual-ring perfect vortices can extend further the application of structured beams in OAM multiplexing for high-capacity and resistant free-space communication [26–28], super-resolution imaging technique [29], and advanced coherent sources [30]. So far, DR-PVs have been obtained using complex optical architectures that basically extended the ones used for perfect vortex generation. Using a binary axicon instead of a linear one, it is possible to generate and shape more diffraction orders obtaining a dual-ring intensity pattern [31–34], while other groups used circular Dammann gratings for the production of equalized concentric rings [35]. Two close rings can also be obtained by the Fourier transform of azimuthally polarized Bessel beams to form a so-called dark PV. However, the sharpness of the dark region degrades with increasing topological charge [36].

Generating the vectorial counterpart requires manipulating the polarization degree of freedom to achieve the superposition of perfect rings with opposite values of orbital and spin angular momenta. In the case of perfect vector beams, that is usually obtained by adding an interferometric stage [37] or additional waveplates and polarizers in cascade. The advent of metasurfaces [38] represented a significant step further in terms of miniaturization and compactness. Benefiting from an unprecedented control on phase, intensity, and polarization [39], metasurfaces can provide the generation of perfect vector beams using a single optical element [40, 41], with a dramatic reduction in complexity and total footprint. Moreover, the design of flat digital optics and their fabrication using the well-established techniques of semiconductor manufacturing open the possibility of integrating the optics directly on chip [42] or on the source [43].

In this work, we present for the first time the realization of dual-functional silicon metaoptics for the compact generation of double-ring perfect vector beams (DR-PVBs) in the telecom infrared. The dual-functional mechanism [44] allows for the spin-decoupled manipulation of a linearly-polarized beam in input in order to upload distinct phase patterns on the two constituent circularly polarized states. Therefore, it is possible to generate non-separable combinations of spin and orbital angular momentum using a single element, imparting at the same time the polarization-insensitive phase pattern required to form the desired double ring without additional optics. In particular, we present two different methods for the computation of the phase patterns, and we apply them to the design of metasurfaces made of digital pillars with different cross-sections and orientations for the control of the dynamic and geometric phases, respectively. The samples are realized using a two-step fabrication protocol, using electron-beam lithography of a resist layer followed by inductively-coupled plasma reactive ion etching (ICP-RIE) to pattern the silicon substrate. Optical characterizations confirm the capability of the optical elements to generate double rings with customized intensity profiles and complex polarization patterns, suggesting novel and smart optical elements for beam shaping with unprecedented levels of compactness and integrability.

## 2 Theory

In this work, we propose for the first time the design, generation, and characterization of particular structured beams called double-ring perfect vector beams (DR-PVBs), using a single full-dielectric metasurface. These beams arise from the merging of two paradigms: vector beams (VBs) and double-ring perfect vortices (DR-PVs). For the benefit of the reader, we summarize in the following paragraphs the theory underlying the manipulation of those beams, describing the numerical methods used in this work for their generation using dual-functional metaoptics.

### 2.1 Vector beams

Vector beams are solutions to Maxwell’s wave equation represented as non-separable combinations of polarization states and spatial modes. They can be mathematically described by introducing a 4-dimensional basis defined by the Cartesian product between a 2D spatial mode basis {|+*ℓ*⟩, |−*ℓ*⟩} and the 2D polarization basis {|*R*⟩, |*L*⟩} [17, 45], where $|R〉={\left[\begin{array}{cc} 1 & i \end{array}\right]}^{T}$ and $|L〉={\left[\begin{array}{cc} 1 & -i \end{array}\right]}^{T}$ are right-handed and left-handed circular polarization states, respectively (the normalization factor $1/\sqrt{2}$ has been omitted), and |±*ℓ*⟩ are *ℓ*-order OAM modes carrying an azimuthal phase term exp(±i*ℓφ*). Actually, the 4D space can also be described as the direct sum of two 2D subspaces, so-called hybrid Poincaré spheres (HPSs) [17]. In particular, the two HPSs include the vortex states (defined by the basis $\left\{u_{R}^{+},u_{A}^{+}\right\}$ ) and the anti-vortex states (defined by the basis $\left\{u_{R}^{-},u_{A}^{-}\right\}$ ), as it follows:

(1) $$\left\{|+\ell 〉,|-\ell 〉\right\}⊗\left\{|R〉,|L〉\right\}=\left\{u_{R}^{+},u_{A}^{+}\right\}⊕\left\{u_{R}^{-},u_{A}^{-}\right\}$$

Then, the general state of an *ℓ*-order vector beam can be described in terms of OAM beams and circular polarization states as:

(2) $$W_{\vartheta ,\chi }^{\pm }=\cos\left(\chi \right){\text{e}}^{-\text{i}\vartheta }|\pm \ell 〉|L〉+\sin\left(\chi \right)e^{+\text{i}\vartheta }|\mp \ell 〉|R〉$$

where the two angles *ϑ* and *χ* refer to the coordinates of the corresponding point on the HPS [46].

As suggested by Eq. (2), a vortex beam can be obtained as the superposition of two coherent beams with opposite spin and OAM using an interferometric setup. The advent of polarization-sensitive metasurfaces allowed improving the compactness of the optical architecture significantly, with a dramatic reduction in the number of required optical elements and the possibility to parallelize the manipulation of the two orthogonal polarization states without the need for a cumbersome interferometric stage [47–49].

### 2.2 Dual-functional metasurfaces

Using dual-functional metalenses, it is possible to impart different optical behaviors to left-handed (LCP) and right-handed (RCP) circular polarizations. That is achieved with a judicious choice of the nanopillars shape and rotation angle in order to control concurrently the dynamic and geometric phases, respectively. Using the Jones formalism, the effect of a metaunit on the polarization basis {|*R*⟩, |*L*⟩} is described by the matrix *J* as

(3) $$J|L〉=-\text{i}{\text{e}}^{\text{i}\left(\delta_{x}+\delta_{y}\right)/2}{\text{e}}^{+\text{i}2\theta }|R〉={\text{e}}^{\text{i}\Phi^{+}}|R〉$$

(4) $$J|R〉=-\text{i}{\text{e}}^{\text{i}\left(\delta_{x}+\delta_{y}\right)/2}{\text{e}}^{-\text{i}2\theta }|L〉={\text{e}}^{\text{i}\Phi^{-}}|L〉$$

being *δ*_*x*_, *δ*_*y*_ the dynamic phases of the given nanopillar, and 2*θ* the geometric phase imparted by a rotation *θ* with respect to the *x*-axis [50, 51]. The equation holds provided the half-wave condition is satisfied, that is, *δ*_*y*_ = *δ*_*x*_ + *π* and the transmission amplitudes are equal along the two directions, *i.e.*, |*T*_*y*_| = |*T*_*x*_|. If we desire that given phase patterns Φ^±^ are transferred, it is sufficient to invert the above relations on the phases to obtain the required maps of dynamic and geometric phases, that is:

(5) $$\delta_{x}=\frac{\Phi^{+}+\Phi^{-}}{2}$$

(6) $$\theta =\frac{\Phi^{+}-\Phi^{-}}{4}$$

Since a general linearly polarized state can be expressed as the superposition of circularly polarized contributions (|*ϑ*⟩ = |*R*⟩e^i*ϑ*^ + |*L*⟩e^−i*ϑ*^) (normalization factor $1/\sqrt{2}$ omitted), the wavefronts of two constituent circularly-polarized terms can be manipulated in parallel to obtain a non-separable combination of polarization and spatial modes as suggested in Eq.(2):

(7) $$J|\vartheta 〉={\text{e}}^{\text{i}\vartheta }{\text{e}}^{\text{i}\Phi^{-}}|L〉+{\text{e}}^{-\text{i}\vartheta }{\text{e}}^{\text{i}\Phi^{+}}|R〉$$

### 2.3 Perfect vortices and perfect vector beams

Vector beams have been originally introduced as linear combinations of Laguerre-Gaussian (LG) beams with opposite spin and orbital angular momenta [17]. However, the ring radius of LG beams is not fixed and increases as the square root of the topological charge (or almost linearly in the case of Kummer beams, generated by spiral phase plates and *q*-plates). The increasing interest in fixed-intensity profiles guided the research on perfect vortices with a customizable ring independent of the carried OAM. Perfect vortices can be obtained in the far field of a Gaussian beam illuminating a spiral axicon, that is, as the Fourier transform of a high-order Bessel–Gaussian beam [15, 16]. Therefore, the cascade of a spiral phase plate and an axicon gives a standard optical architecture, followed by a lens in *f–f* configuration. On the back-focal plane of the lens, an intensity ring is obtained with radius and width given by *R* = *αf*/*k* and Δ*R* = 2*f*/(*kW*), respectively, being *W* the waist of the input Gaussian beam, and *α* the axicon parameter. While diffractive optics and spatial light modulators substituted the initial refractive optics with their flat counterparts, it was only with metasurfaces that the highest integration level was reached, especially for generating perfect vortex beams. Then, we define:

(8) $$\Phi^{\pm }=\Omega_{\pm \ell }+\Omega_{\text{axicon}}+\Omega_{\text{lens}}$$

being Ω_±*ℓ*_ = ±*ℓφ* the staircase profiles mandatory to transfer ±*ℓ* OAM per photon in units of *ℏ* to the impinging beam [52], Ω_axicon_ = −*αr* the phase profile of an axicon [53], and $\Omega_{\text{lens}}=-k\left(\sqrt{r^{2}+f^{2}}-f\right)$ an aberration-free focusing phase profile with focal length *f* at *k* = 2*π*/*λ* [54]. Inserting Eq. (8) in Eq. (7), we obtain a dual-functional metalens generating perfect vector beams on its focal plane.

### 2.4 Generation of double-ring perfect vector beams

Double-ring perfect vortices are beams endowed with two closely located rings having the same intensity peak and carrying orbital angular momentum. Moreover, as the optical vortices are “perfect”, the diameters of the two rings should be independent of the carried topological charge.

In this work, we present two different solutions to generate double-ring perfect vector beams, *i.e.*, double-ring perfect vortices with non-separable combinations of spin and OAM. Based on high-order associated Laguerre polynomials, the first method is suitable only for generating double-ring perfect vortices with the same vectorial order. Conversely, the second one encodes two equalized spiral axicon terms and can be used to generate rings with arbitrary vectorial order.

From the literature, it is suggested that the phase function of a phase-only element able to generate high-order LG beams is [55, 56]:

(9) $$\Omega_{p}^{\ell }={\text{e}}^{\text{i}\ell \phi }\cdot \mathrm{sign}\left[L_{p}^{\left|\ell \right|}\left(\frac{2r^{2}}{w_{0}^{2}}\right)\right]$$

where *p* is the radial index, corresponding to the number of zeros of the associated Laguerre polynomial $L_{p}^{\left|\ell \right|}$ , *w*_0_ is the beam waist radius of the LG beam. Equation (9) introduces a number *p* of radial *π* discontinuities to the staircase phase profile. For the sake of simplicity, we define $c_{p}^{\left|\ell \right|}=\mathrm{sign}\left[L_{p}^{\left|\ell \right|}\left(2r^{2}/w_{0}^{2}\right)\right]$ and we introduce it in Eq. (8) imposing *p* = 1:

(10) $$\Phi^{\pm }=c_{1}^{\left|\ell \right|}\Omega_{\pm \ell }+\Omega_{\text{axicon}}+\Omega_{\text{lens}}$$

obtaining a new phase profile that encodes the Fourier transform (given by the lens profile) of a high-order Bessel–Gaussian beam (given by the combination of an axicon and a high-order staircase profile) that produces a double-ring perfect optical vortex carrying |*ℓ*| orbital angular momentum [57]. Finally, the dual-functional paradigm is exploited to design a metalens for the generation of double-ring perfect vector beams, by combining Eqs. (7) and (10), and recalling the relationship between a general linear polarization state and its circularly-polarized components.

However, in case different orders are required for the two rings, the previous approach is not suitable due to the intrinsic property of Eq. (9) to impart the same OAM both to the inner and outer rings. To overcome this issue, we propose a second method based on an equalized double axicon. Recalling Eq. (8) for the generation of a perfect vortex, it can be easily noticed that by varying the axicon parameter *α* it is possible to change the diameter of the ring [57]. Therefore, if we could generate two non-overlapping PVs carrying different OAM using the same pattern, we would satisfy the request for different vectorial orders. To this aim, we introduce a new equation for the phase patterns Φ^±^ of the dual-functional metalens:

(11) $$\Phi^{\pm }=\arg\left(\overset{2}{\underset{i=1}{\sum }}A_{i}\cdot {\text{e}}^{\text{i}\Omega_{i}^{\pm }}\right)$$

where *i* = 1, 2, *A*_*i*_ are the different weights for each perfect vortex, and

(12) $$\Omega_{i}^{\pm }=\Omega_{\pm \ell_{i}}+\Omega_{\text{axicon,i}}+\Omega_{\text{lens}}$$

In particular, to obtain a double ring, we need to impose the same focal length but different spatial frequencies for the two axicon terms that is *α*_1_ ≠ *α*_2_. Therefore, by controlling the difference between the two parameters, we can tune the distance between the two intensity rings. Conversely, by choosing different *ℓ*_*i*_ (*i* = 1, 2), we can set the orders of the two concentric perfect vector beams arbitrarily.

In the following sections, we show the simulation, fabrication, and optical characterization of a few dielectric metaoptics designed for generating different DR-PVBs using the two above-mentioned techniques.

## 3 Design and characterization

### 3.1 Simulations

To implement the dual-functional metalens paradigm for wavefront engineering, it is mandatory to find the set of optimized metaunits filling the whole metalens area. We performed custom finite element method (FEM) simulations (COMSOL Multiphysics) at the working wavelength of 1310 nm to extrapolate the geometric features of each metaunit. The period of the metaatoms matrix was fixed at 600 nm in order to satisfy the subwavelength regime, while we let the sizes of the metaunit cross-section sweep, after fixing the height at 850 nm. Thus, for each metaunit we selected the cross sections satisfying the half-wave plate (HWP) condition Δ = *π*, where Δ = *δ*_*x*_ − *δ*_*y*_, being *δ*_*x*_ and *δ*_*y*_ the phase delays for linear polarizations parallel to the *x*- and *y*-axis, respectively. In addition, we imposed strict conditions to ensure total polarization conversion, as shown in Eqs. (3) and (4), and a homogeneous transmittance over the whole metalens. More precisely, we selected metaatoms having at the same time a maximum deviation of 0.03 rad from the HWP condition, a difference in transmission Δ_*T*_ = |*T*_*x*_ − *T*_*y*_| lower than 0.05, and satisfying a maximum deviation of 0.1 in terms of average transmission (*T*_AVG_ = (*T*_*x*_ + *T*_*y*_)/2) among the simulated metaatoms.

As a result of the previous requirements, a meta-library of 13 different nanopillars with an average transmission of 0.75 has been extrapolated, which permits a well-distributed 13-level discretization of the phase over the whole range 0–2*π*. Conversely, we assumed no discretization on the geometric phase [58]. Then, for given phase patterns Φ^±^, we could calculate the corresponding maps of the dynamic and geometric phases, providing the recipe to compute the metaatoms pattern for the desired metaoptics. The optical response was simulated using a custom MatLab^®^ code implementing the Fresnel propagator [59] over a squared computational window with a side of 600 μm and pixel size of 600 nm. Simulations considered metalenses of radius 300 μm, designed at the working wavelength of 1310 nm with a dynamic phase discretization over 13 levels and illuminated by a Gaussian beam with a waist of 300 μm to cover the metasurface area adequately and avoid boundary effects.

### 3.2 Fabrication

**Figure 1:**
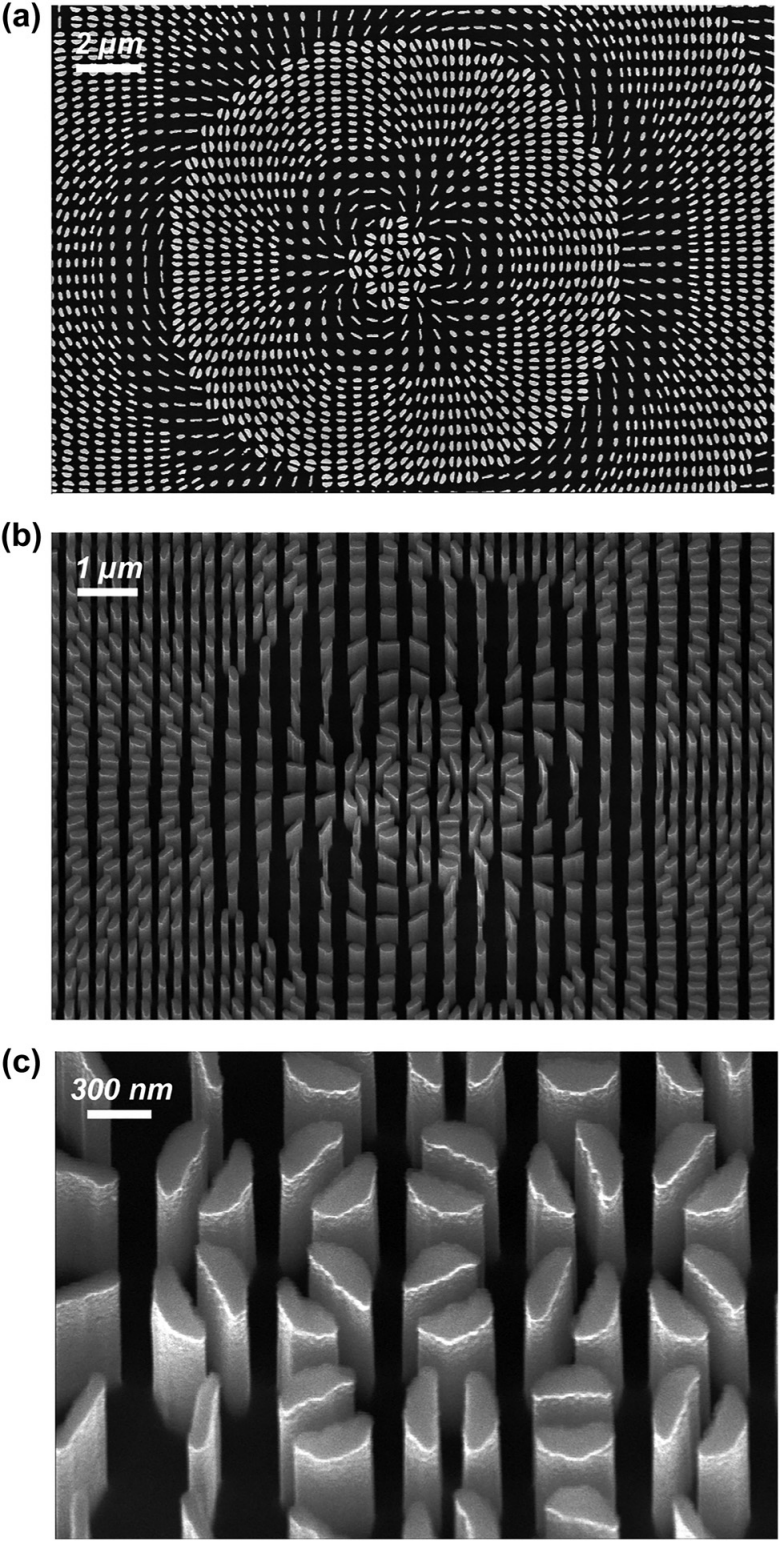
SEM inspection of a fabricated sample. (a) Top view and (b) tilted view where the different cross-sections and rotation of the nanopillars are appreciable. (c) Detailed zoom of the metalens central area.

The designed metaoptics were realized using a two-step fabrication protocol. We used electron beam lithography (EBL) to transfer the optimized computational pattern onto the physical sample [60, 61]. A thin PMMA resist layer deposited on ⟨100⟩ silicon substrates has been patterned using an EBL system (Carl Zeiss Sigma 300, 30 keV beam voltage). Then, an alumina mask was deposited after development by means of electron gun evaporation of an Al_2_O_3_ target in high vacuum conditions. Following deposition, a lift-off process through sonication in hot acetone left the Al_2_O_3_ on the previously exposed regions. Finally, the pattern was transferred onto the underlying silicon substrate through inductively coupled plasma reactive ion etching (ICP-RIE). All the fabricated metasurfaces had a diameter of 600 μm. In Figure 1, a few SEM images of one of the fabricated silicon metasurfaces are reported.

### 3.3 First method: high-order LG beam

**Figure 2:**
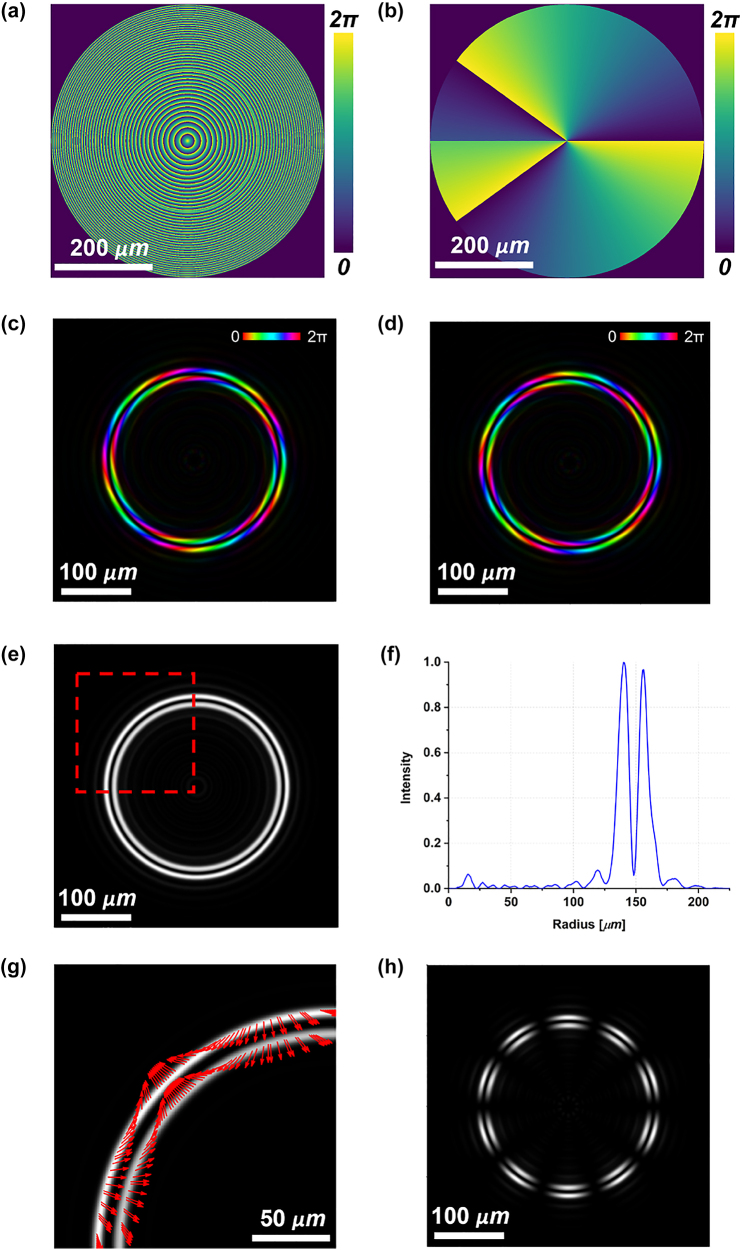
Simulations of double-ring perfect vector beams (DR-PVBs) generation using high-order LG beam technique. (a) Dynamic phase map *δ*_*x*_ and (b) local nanopillars rotation angle *θ* required to impart the geometric phase contribution 2*θ*. Phase and amplitude distributions of the scalar DR-PVs generated under LCP (c) or RCP (d) light in input. Brightness and colors refer to intensity and phase, respectively. Intensity profile (e) and integrated radial profile (f) of the DR-PVB generated with vertically polarized light in input. (g) Polarization plot of the upper-left quarter of the beam marked in (e). (h) Petal-like intensity configuration obtained after filtering the beam in (e) with a linear polarizer oriented along the horizontal direction.

We started designing a metasurface for the generation of a double-ring perfect vector beam using the high-order LG beam technique. Recalling Eq. (10), we imposed the axicon parameter (*α*) equal to 0.383 and the focal length (*f*) equal to 1.5 mm for both the phase patterns Φ^+^ and Φ^−^. Conversely, we chose opposite values of *ℓ* (*i.e.*, *ℓ*^+^ = 5 and *ℓ*^−^ = −5) to obtain a vector beam when a linearly polarized light impinges on the metaoptics. In order to equalize the two rings and ensure a dark region in between, the parameter *w*_0_ in Eq. (9) must be carefully optimized, depending on the beam waist *W* in input. Figure 2(a) and (b) show the dynamic phase and the metaunits rotation angle. As clearly visible in the dynamic phase (Figure 2(a)), only one *π* phase discontinuity, typical of a first-order Laguerre–Gaussian mode, has been introduced [55]. In Figure 2(c) and (d), the intensity and phase maps of the scalar DR-PVs generated for impinging LCP and RCP beams are shown, respectively. It can be noticed that, depending on the handedness of the polarization state in input, the two rings carry opposite values of OAM, as expected. The intensity map of the vector beam generated using an impinging linear polarization state is shown in Figure 2(e). Figure 2(f) and (g) depict the radial profile and the upper-left quarter polarization state of the generated beam, respectively. Finally, Figure 2(h) shows the expected 2|*ℓ*|-petal pattern generated by vertically polarized light after applying a filtering polarizer with the principal axis orthogonal (*i.e.*, horizontal direction) to the input polarization state, which confirms the vectorial nature of the generated double ring. As expected, the two rings have the same vectorial order and the same intensity profile (Figure 2(g)) as required by the imposed constraints.

**Figure 3:**
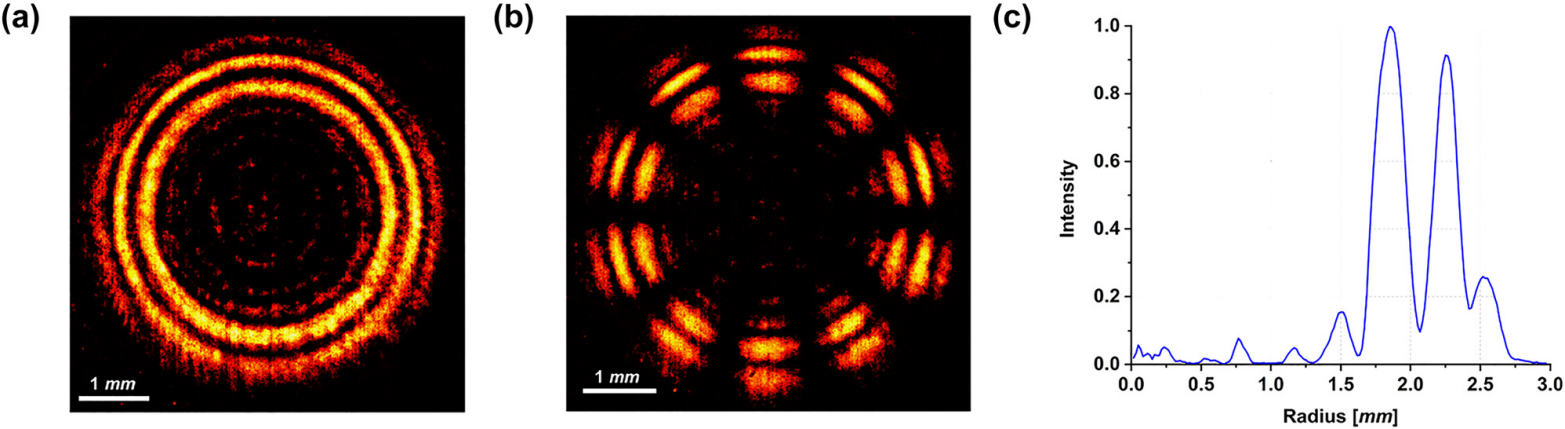
Optical characterization of the sample designed in Figure 2. (a) Experimental DR-PVB generated by the metasurface designed using the high-order LG method and illuminated with vertically polarized light. (b) Corresponding petal-like configuration measured using a linear polarizer as analyzer. (c) Integrated radial profile. The measured results are consistent with those simulated in Figure 2.

**Figure 4:**
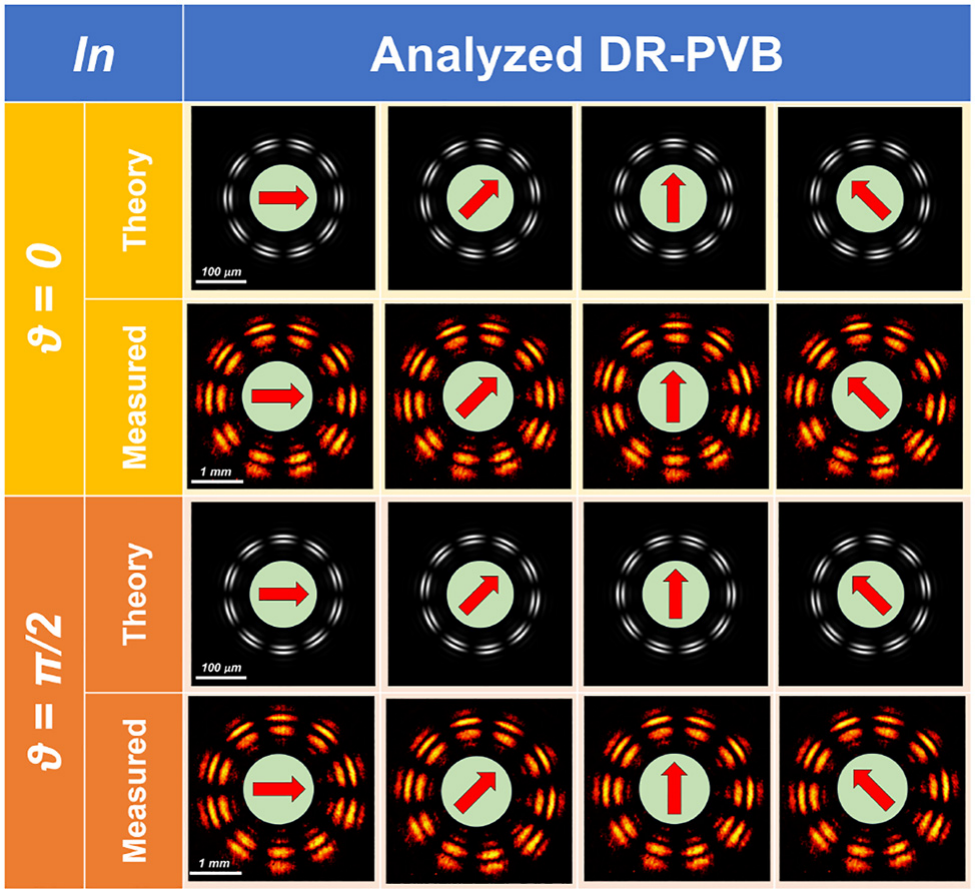
Generation of DR-PVBs using the high-order LG method and analysis using a rotating linear polarizer in cascade. In the first two rows (in yellow), we have horizontally polarized light in input and the figures show the corresponding simulated and measured intensity profiles as a function of the analyzer orientation (depicted by using a red arrow on a green circle). In the third and fourth rows (in orange) we have vertically polarized light in input. Both the inner and outer rings are 5-th order perfect vector beams inducing associated 10-petal intensity profiles after the analyzer. It is clearly visible that the counter-clockwise rotation of the intensity pattern is associated with a counter-clockwise rotation of the analyzer.

Figure 3(a) shows the experimental intensity pattern of the generated beam obtained with vertically polarized light (*ϑ* = *π*/2) illuminating the fabricated metaoptics. It must be noticed that the measured DR-PVB is about 10 times the size of the simulated one, since a 10× objective was used to illuminate the whole area of the acquisition camera. In Figure 3(b) the beam in Figure 3(a) has been filtered using a linear polarizer with its transmitting axis rotated by *π*/2 degrees, exhibiting the expected 10-petal constellation shown in Figure 2(h). Figure 3(c) depicts the radial profile of the intensity pattern in (a), confirming that the two rings are almost equalized and a dark zone is present in between. Finally, we characterized the DR-PVB analyzing all the configurations assumed under different impinging polarizations and varying the analyzer rotation (Figure 4). It is worth noting that the measured results perfectly agree with the simulations.

### 3.4 Second method: equalized double axicon

To test the flexibility of this second technique, we designed and characterized two different metaoptics. The first sample can generate the same DR-PVB described in Section 3.3 but with the possibility of tuning the dark zone between the two rings. Recalling Eq. (11) and the underlying theory described in Section 2.4 we impose *ℓ*_1_ = *ℓ*_2_ = 5 to Φ^+^ and Φ^−^ in order to obtain a 5th order vector beam. Moreover, to tune the dark zone and the ring equalization, we must carefully select the parameters {*α*_*i*_, *A*_*i*_}. We chose *α*_1_ = 0.383 and *α*_2_ = 0.465 to well separate the two rings. Then, we iteratively optimized the choice of *A*_1_ and *A*_2_ to equalize the ring intensities. It is worth noting that *A*_2_ > *A*_1_ because we need to compensate for the energy spreading due to the larger radius of the outer ring.

**Figure 5:**
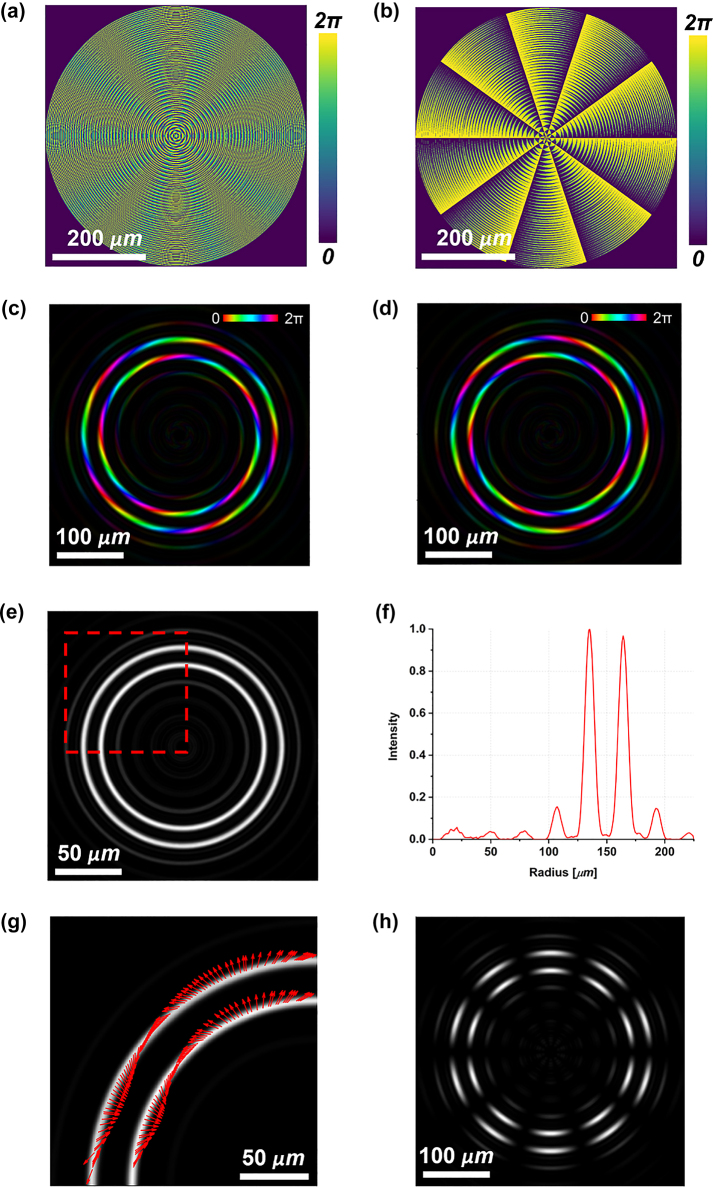
Simulations of double-ring perfect vector beams (DR-PVBs) generation having rings with the same vectorial order, by using the equalized double axicon method. (a) Dynamic phase map *δ*_*x*_ and (b) local nanopillars rotation angle *θ* required to impart the geometric phase contribution 2*θ*. Phase and amplitude distributions of the scalar DR-PVs generated under LCP (c) or RCP (d) light in input. Brightness and colors refer to intensity and phase, respectively. Intensity profile (e) and integrated radial profile (f) of the DR-PVB generated with vertically polarized light in input. It can be observed that the dark region between the two rings is larger than the one generated using the high-order LG method and depicted in Figure 2. (g) Polarization plot of the upper-left quarter of the beam marked in (e). (h) Petal-like intensity configuration obtained after filtering the beam in (e) with a linear polarizer oriented along the horizontal direction.

**Figure 6:**
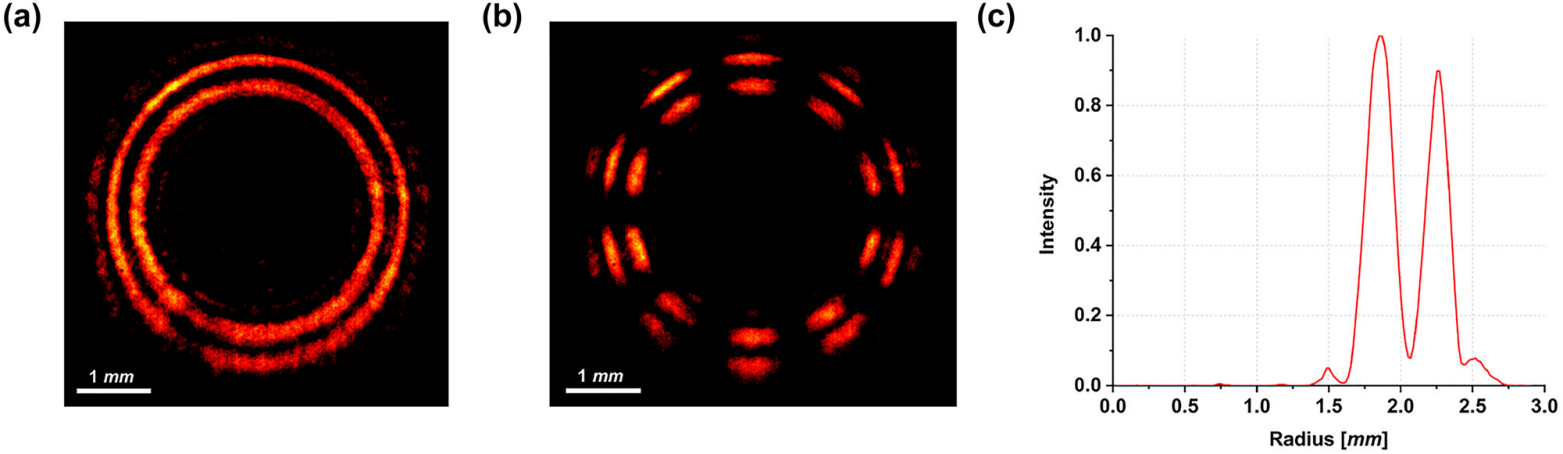
Optical characterization of the sample designed in Figure 5. (a) Experimental DR-PVB generated by the metasurface designed using the double axicon method and illuminated with vertically polarized light. (b) Corresponding petal-like configuration measured using a linear polarizer as analyzer. (c) Integrated radial profile. The measured results are consistent with those simulated in Figure 5.

Figure 5(a) and (b) depict the dynamic phase and the rotation angle over the whole metasurface area, respectively. It is worth noting that these phase profiles are completely different from the ones encoded using the high-order LG method (Figure 2(a) and (b)), but in both cases the same DR-PV is generated as depicted in Figures 2(c)–(f) and 5(c)–(f). As described above, a benefit of the equalized double axicon approach is the possibility of tuning the dark region. In fact, it is clearly visible from the radial profiles that the dark region between the two rings is larger in Figure 5 with respect to Figure 2. Then, the DR-PVB generated by the fabricated metasurface has been characterized under linearly polarized impinging light. Also in this case, two equalized rings with a vectorial nature are formed, as confirmed by the petal-like intensity patterns (Figure 6).

**Figure 7:**
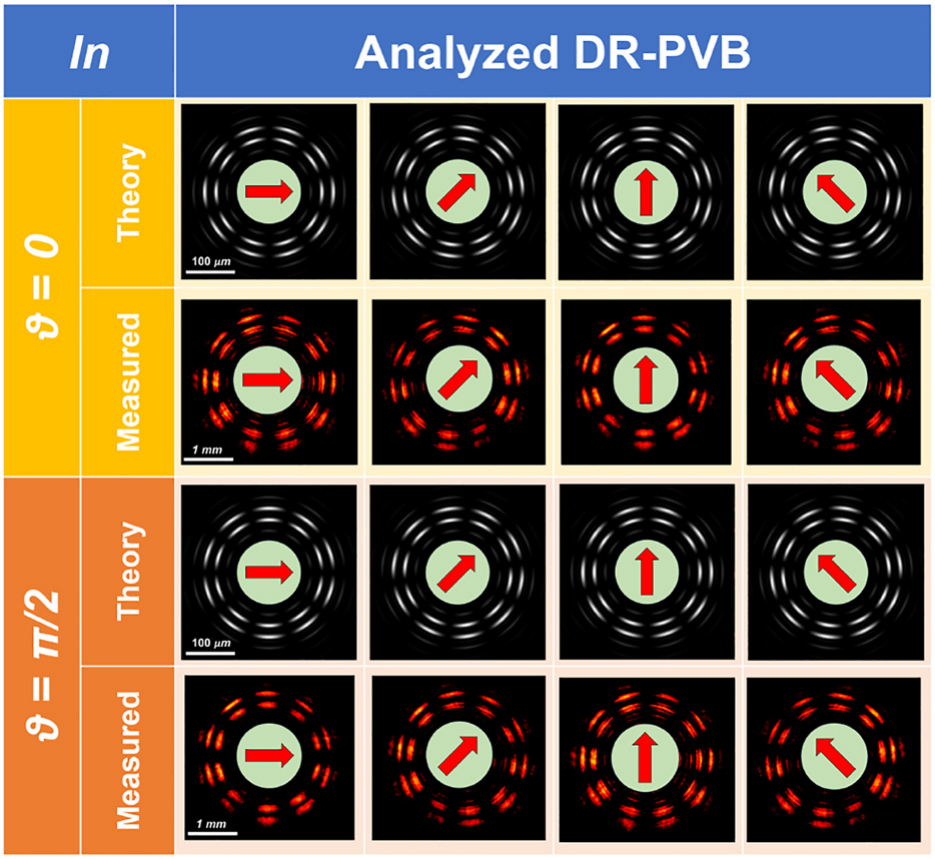
Generation of DR-PVBs (having the same vectorial order for both the inner and the outer ring) and analysis using a rotating linear polarizer in cascade. In this case the double axicon technique was used. In the first two rows (in yellow), we have horizontally polarized light in input and the figures show the corresponding simulated and measured intensity profiles as a function of the analyzer orientation (depicted by using a red arrow on a green circle). In the third and fourth rows (in orange) we have vertically polarized light in input. Both the inner and outer rings are 5th order perfect vector beams inducing associated 10-petal intensity profiles after the analyzer. It is clearly visible that the counter-clockwise rotation of the intensity pattern is associated with a counter-clockwise rotation of the analyzer.

Thus, we explored all the vectorial configurations by changing the linear polarization state in input and rotating the analyzer. The experimental results perfectly agree with the simulated ones (Figure 7).

The second metalens is able to generate DR-PVB having rings with different vectorial orders. Recalling Eq. (11), we impose *ℓ*_1_ = 1 and *ℓ*_2_ = 2 in order to obtain a 1st order vector beam for the inner ring and a 2nd order vector beam for the outer one. For simplicity, we used the same {*α*_*i*_} parameters (*i.e.*, *α*_1_ = 0.383 and *α*_2_ = 0.465) and equalization parameters (*i.e.*, *A*_1_ and *A*_2_) to generate the DR-PVB with equalized and well-separated rings.

**Figure 8:**
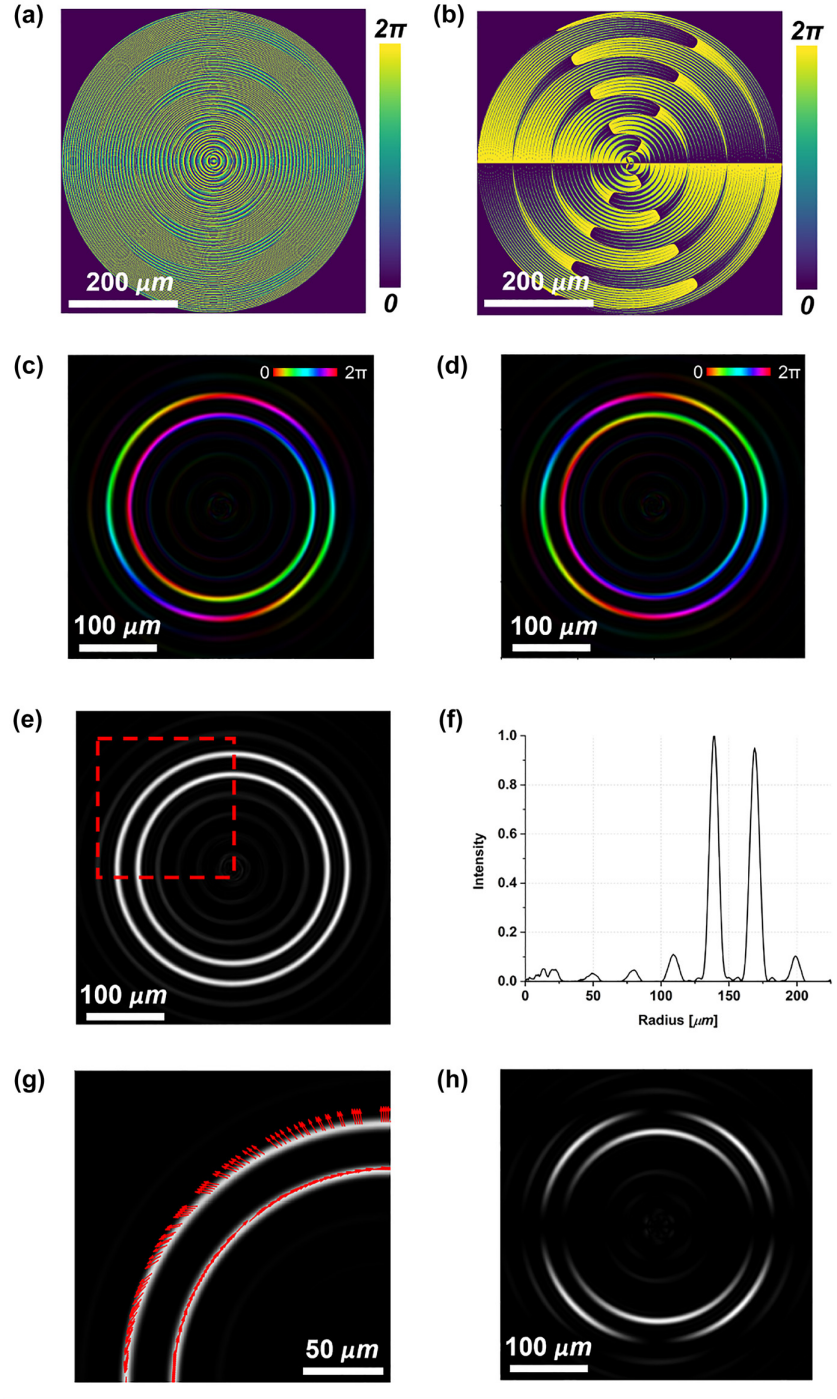
Simulations of the double-ring perfect vector beams (DR-PVBs) generation having rings with different vectorial orders, by using the equalized double axicon method. (a) Dynamic phase map *δ*_*x*_ and (b) local nanopillars rotation angle *θ* required to impart the geometric phase contribution 2*θ*. Phase and amplitude distributions of the scalar DR-PVs generated under LCP (c) or RCP (d) light in input. Brightness and colors refer to intensity and phase, respectively. The inner radius carries either +1 or −1 OAM, conversely the outer one carries either +2 or −2 OAM, depending on the input handedness. Intensity profile (e) and integrated radial profile (f) of the DR-PVB generated with vertically polarized light in input. (g) Polarization plot of the upper-left quarter of the beam marked in (e). (h) Petal-like intensity configuration obtained after filtering the beam in (e) with a linear polarizer oriented along the horizontal direction. As expected, a different number of petals is generated between the two rings, depending on their vectorial order.

**Figure 9:**
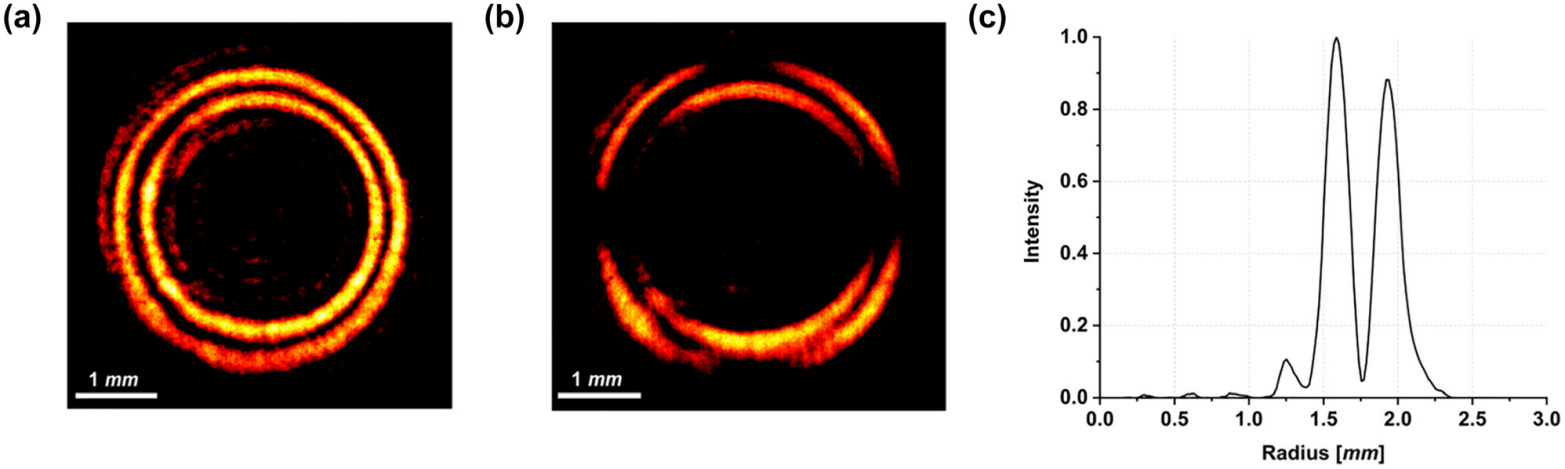
Optical characterization of the sample designed in Figure 8. (a) Experimental DR-PVB generated by the metasurface designed using the double axicon method and illuminated with vertically polarized light. (b) Corresponding petal-like configuration measured using a linear polarizer as analyzer. (c) Integrated radial profile. The measured results are consistent with those simulated in Figure 8.

**Figure 10:**
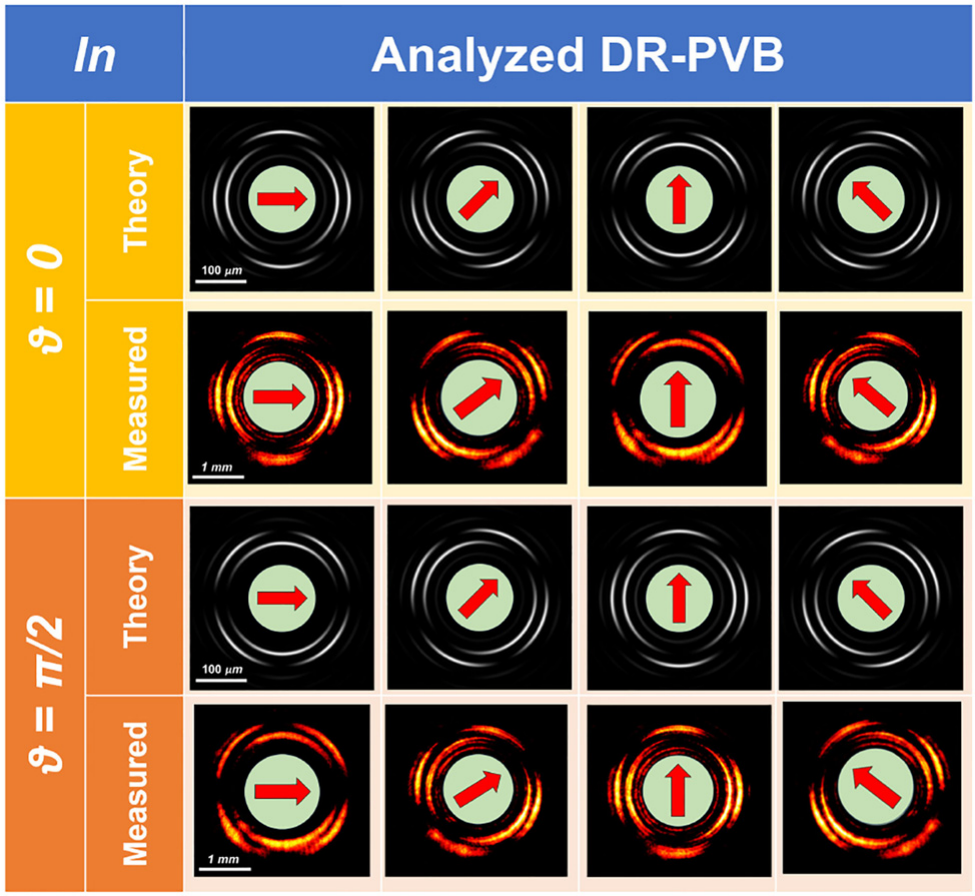
Generation of DR-PVBs with different vectorial order between the inner and the outer rings and analysis using a rotating linear polarizer in cascade. In this case the double axicon technique was used. In the first two rows (in yellow), we have horizontally polarized light in input and the figures show the corresponding simulated and measured intensity profiles as a function of the analyzer orientation (depicted by using a red arrow on a green circle). In the third and fourth rows (in orange) we have vertically polarized light in input. The inner ring is a 1st order perfect vector beams with an associated 2-petal intensity profile after the analyzer. The outer one is a 2nd order perfect vector beam with an associated 4-petal intensity profile. It is clearly visible that the counter-clockwise rotation of the intensity pattern is associated with a counter-clockwise rotation of the analyzer.

In Figure 8, all the simulation results are collected. In particular, Figure 8(c) and (d) show the spin-dependent response of the metaoptics with the two rings carrying different OAM, while Figure 8(e) and (f) depict the peculiarities of the generated DR-PVB. Figure 9 shows the experimental results using the fabricated metalens under linearly polarized light in input. It can be easily noticed that the two rings are well equalized and separated, while they have different vectorial orders (*i.e.*, 2 petals for the inner ring and 4 for the outer one). Finally, we characterized all the vectorial configurations as in the previous cases. Again, the experimental results are in good accordance with the simulations (Figure 10).

## 4 Discussion

Double-ring perfect vector beams provide a unique combination of OAM-independent profile, vectorial nature, and multi-ring intensity distribution. Benefiting from the combined manipulation of polarization and phase offered by dual-functional metaoptics, we exploited the parallel optical processing of left-handed and right-handed circularly polarized states to combine spatial modes having opposite values of spin and OAM in a non-separable manner for the generation of vector beams. In particular, we introduced and tested two alternative techniques for encoding a customized double-ring perfect profile. Based on high-order Laguerre–Gaussian beams, the first method introduces a *π* radial discontinuity in the phase pattern and is suitable for generating close rings with the same vectorial order. The latter one, based on double axicon encoding, allows also tuning the size of the dark region in between and imparting different orders to the two rings. In principle, the same techniques can be generalized to any number of concentric perfect vortices with customized ring sizes and topological charges.

Although the library of silicon metaatoms has been optimized for the wavelength of 1310 nm, the designed metalens is expected to exhibit high values of efficiency conversion and transmission over the whole telecom O-band between 1260 and 1360 nm [58] exploiting the transparency of silicon in this range. By further engineering the cross-section, it could be possible to also control the group delay and group delay dispersion of the metaatoms [62] in order to compensate for chromatic aberration, achieving the same focal length independently of the input wavelength. Analogous metaoptics can be designed for the visible range, patterning a layer of amorphous silicon deposited over a glass substrate or moving to a different high-index dielectric material such as titanium dioxide, provided the calculation of a new set of metaatoms is performed.

## 5 Conclusions

Double-ring complex beams represent a smart solution for the optical manipulation of low refractive index particles. In this work, we presented the design, fabrication, and test of a new class of full-dielectric metaoptics for the compact generation of novel structured beams with spatially-variant intensity, polarization, and phase.

The possibility to generate such beams using a single metasurface dramatically simplifies the optical path complexity in the number of optical elements and optical processing stages, with a significant improvement in efficiency, alignment, costs, and miniaturization. These optics and their ability to generate beams with two concentric rings having a vectorial nature suggest compact and integrated optical solutions for optical trapping, tweezing, and manipulation in a microfluidic environment. Concurrently, the same beams can find promising applications for trapping cold atoms in optical processing and quantum computing or for exploiting complex light patterns for high-capacity communications and light–matter interaction.

## Supplementary Material

Supplementary Material Details
