# Supplementary material for: All-dielectric metaoptics for the compact generation of double-ring perfect vector beams
Source: Nanophotonics. 2023 Oct 30;12(22):4215–28. doi: 10.1515/nanoph-2023-0555 (PMC11501850; doi:10.1515/nanoph-2023-0555)
Supplement: Supplementary file 1 — Supplementary Material Details [file j_nanoph-2023-0555_suppl_001.pdf]

Supplementary Material

Andrea Vogliardi, Gianluca Ruffato*, Daniele Bonaldo, Simone Dal Zilio, and Filippo Romanato

All-dielectric metaoptics for the compact generation of double-ring perfect vector beams

S1 Metaatoms list extraction

We set up custom-made Finite-Element Method (FEM) numerical simulations in the wavelength domain (using COMSOL Multiphysics®) to find the best set of metaatoms satisfying the requirements imposed by the half-wave plate condition. Each subunit has been defined as a silicon nanopillar (n_{Si}=3.5030) surrounded by air (n_{air}=1) placed on the top of a silicon substrate. All the materials were considered as non-absorbing in the range under study ($n = Re(n)$, $Im(n) = 0$). Thus, we imposed some conditions to simulate the nanostructures properly: Periodic Port conditions were set in the substrate at a distance equal to λ below the nanopillar and at a distance greater than λ above it, both to collect the scattering parameters of the structure and simultaneously ensure the far-field regime [1]. Perfectly Matched Layer (PML) conditions have been imposed outside the ports at a distance greater than λ to visualize the transmitted and reflected fields, and to absorb the field over a certain distance to avoid unwanted multiple reflections. Finally, Periodic Boundary Conditions (PBC) were set to permit the correct simulation of the interaction between the various metaunits of the metasurface [2]. Among all the possible solutions, a subset of 13 pillars (Figure S1) was selected covering the whole 2π range of dynamic phase and satisfying the conditions of half-wave plate and equal transmissions for x and y polarizations within the tolerance described in **3.1 Simulations**[3].

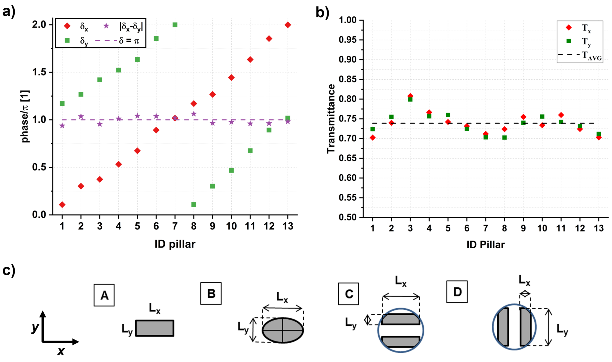

d)

ID	1	2	3	4	5	6	7	8	9	10	11	12	13
Type	C	C	C	B	A	D	D	D	D	B	A	C	C
L_x [nm]	400	350	425	400	480	190	180	160	150	190	100	500	400
L_y [nm]	160	150	120	170	100	500	400	400	350	380	480	190	180

Fig. S1: Library of different silicon nanopillars working at 1310 nm providing the recipe to build up a dual-functional metasurface. (a) Phase delays for y and x polarizations, (b) Transmittance for y and x polarizations (c) Different types of pillars composing the metalibrary. Rectangular and elliptical pillars (A-B) are more common, with respect to the other two types (C-D) made of two paired pillars, with a rectangular base inscribed within a concentric circle of radius 250 nm and placed at a distance of 300 nm between the two centers. (d) Complete list of the metaatoms library showing the type of pillars and the corresponding size.

S2 Anti-vortex states generation

Vector beams are described as the coherent superposition of two circularly polarized beams with opposite spin and different spatial configurations:

$$W^{\pm}_{\vartheta,\chi} = cos(\chi)e^{-i\vartheta}|\pm\ell\rangle|L\rangle + sin(\chi)e^{+i\vartheta}|\mp\ell\rangle|R\rangle \quad (1)$$

It is worth noting that by adding a half-wave plate in cascade, it is possible to switch the handedness of the circularly polarized contributions and bounce between

Andrea Vogliardi, Department of Physics and Astronomy 'G. Galilei', University of Padova, via Marzolo 8, 35131, Padova, Italy; E-mail: vogliardi.andrea@unipd.it; https://orcid.org/0000-0002-8864-506X
***Corresponding author: Gianluca Ruffato,** Department of Physics and Astronomy 'G. Galilei', University of Padova, via Marzolo 8, 35131, Padova, Italy; E-mail: gianluca.ruffato@unipd.it; https://orcid.org/0000-0003-3997-0488
Daniele Bonaldo, Department of Information Engineering, University of Padova, via Gradenigo 6, 35131, Padova, Italy
Simone Dal Zilio, CNR-IOM Istituto Officina dei Materiali, S.S. 14 - Km. 163,5 – 34149, Trieste, Italy
Filippo Romanato, Department of Physics and Astronomy 'G. Galilei', University of Padova, via Marzolo 8, 35131, Padova, Italy; https://orcid.org/0000-0003-0243-1464

the two hybrid Poincaré spheres (from the "vortex" state to the corresponding "anti-vortex" one) [3].

For the sake of completeness, we have characterized the behaviour of the fabricated metasurfaces for the generation of double-ring perfect antivortex beams with two different vectorial orders between the two rings. As shown in the main manuscript for the vortex states, a counter-clockwise rotation of the intensity profile is associated with a counter-clockwise rotation of the analyzer. On the contrary, the rotation of the intensity pattern is clockwise in the case of anti-vortices, as expected from the theory and shown in Fig. S2.

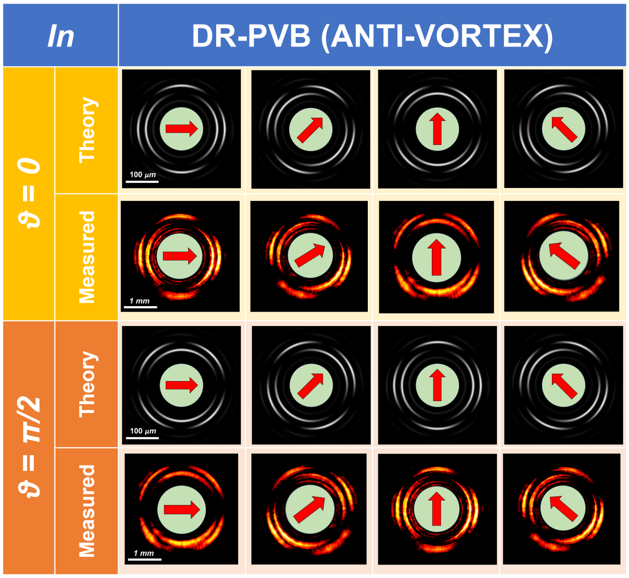

Fig. S2: Generation of double-ring perfect anti-vortices with different vectorial order between the inner and the outer rings and analysis using a rotating linear polarizer in cascade. The double axicon technique was used. In the first two rows (in yellow), we have horizontally polarized light in input and the figures show the corresponding simulated and measured intensity profiles as a function of the analyzer orientation (depicted by using a red arrow on a green circle). In the third and fourth rows (in orange) we have vertically polarized light in input. The inner ring is a 1-st order perfect vector beam with an associated 2-petal intensity profile after the analyzer. The outer one is a 2-nd order perfect vector beam with an associated 4-petal intensity profile. It is clearly visible that the clockwise rotation of the intensity pattern is associated with a counter-clockwise rotation of the analyzer, which is the opposite behaviour of the vortex states shown in the main manuscript.

S3 Optical Characterization

The optical behaviour of the fabricated metalens has been tested using the experimental setup depicted in Figure S3. The input Gaussian beam of the desired waist was generated with a (Liquid Crystal on Silicon) LCoS spatial light modulator (SLM) (X13267-08, Hamamatsu, pixel pitch 12.5 μm) using a phase and amplitude modulation technique [4]. The output of a DFB laser (λ = 1310 nm, 1310LD34 1-2-2-1 CCSI, AeroDiode) was collimated at the end of a single mode fiber using an aspheric lens with focal length $f_F = 7.5mm$ (A375TM-C, Thorlabs), linearly polarized (LPIREA100-C, Thorlabs) and expanded by a first telescope ($f_1 = 3.5cm$, $f_2 = 10.0cm$) before illuminating the display of the SLM. Then, a 4-f system ($f_3 = 20.0cm$, $f_4 = 12.5cm$) with an aperture in the Fourier plane was used both to isolate the first-order encoded mode and adjust the beam waist to $W = 300\mu m$. A 50:50 beam-splitter was placed before the SLM to send the reflected beam into the desired optical path. In between, a quarter-wave plate (QWP) (WPQ10M-1310, Thorlabs) or a half-wave plate (HWP_1 in Figure S3) (WPH05M-1310, Thorlabs) were used to set the desired polarization state. In the specific, the QWP was exploited to generate circularly polarized states from the original horizontally polarized one, while the HWP was used, alternatively, to rotate the polarization plane of the beam exiting the SLM and generate the desired vector beam. The polarized Gaussian beam with the desired size illuminated the patterned zone of the metaoptics, mounted on a 6-axis kinematic mount (K6XS, Thorlabs). A 10x Objective (CFI E Plan Achromat 10X, Nikon) mounted on a micrometric translator stage (LX20/M, Thorlabs) was used to collect the output beam. The removable sequence of a second HWP and an additional linear polarizer was used to select on demand the anti-VB basis (HWP_2 in Figure S3) and to analyze the VB configuration (P_2), respectively. A camera (WiDy SWIR 640U-S, pixel pitch 12.5 μm) was used to image the collected beam. The different scale is related to the magnification factor introduced in the experimental setup. A microscope objective is necessary to extract and magnify the generated beams properly. As a matter of fact, the focal length of 1.5mm is too short to collect the beam directly on the CCD camera. Moreover, the beam would be better appreciable exploiting the whole sensitive area of the camera. This explains the different scale bars between simulations and experi-

mental acquisitions in the figures. Finally, we measured the experimental efficiency of our metaoptics obtaining an average transmission value of 72% which is close to the theoretical value (75%).

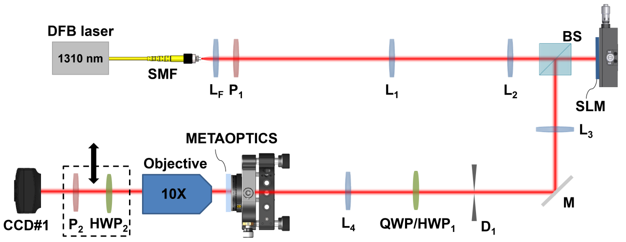

Fig. S3: Layout of the experimental setup. The output of a DFB laser at $1310nm$ is collimated (aspheric lens L_F), linearly polarized (polarizer P_1), and expanded (lenses L_1, L_2) before illuminating a LCoS SLM for beam shaping. A 50:50 beam-splitter (BS) is used to redirect the reflected beam. The resized Gaussian beam is filtered using a $4f$ setup (L_3, L_4, D_1) and illuminates the sample placed on a 6-axis kinematic mount. The input polarization state is controlled using either a quarter-wave plate (QWP) or a half-wave plate (HWP_1). The output beam is expanded using a 10X objective and detected with a camera. A second half-wave plate can be inserted or removed to generate anti-vortex states. A rotating polarizer (P_2) is used for vector beams analysis.

S4 Generation of DR-PVBs with different vortex states

The generation of DR-PVBs using the equalized double axicon method proposed in **2.4 Generation of double-ring perfect vector beams** is very flexible and also permits the encoding of different "vortex states" on the two rings. For the benefit of the reader, we designed a metaoptics that generates a 1-st order anti-vortex state in the outer ring and a 5-th order vortex state in the inner ring. In this regard, we imposed:

$$\Omega_1^{\pm} = \Omega_{\pm 5} + \Omega_{axicon,1} + \Omega_{lens} \tag{2}$$

$$\Omega_2^{\pm} = \Omega_{\mp 1} + \Omega_{axicon,2} + \Omega_{lens} \tag{3}$$

choosing the same focal length ($f = 1.5mm$) and the axicon parameters $\alpha_1 = 0.383$ and $\alpha_2 = 0.520$ in order to generate the desired DR-PVBs as depicted in Figure S4. To confirm the generation of different vector states, we simulated the DR-PVBs analyzing all the configurations assumed under different impinging polarization and varying the analyzer rotation (Figure S5). It is worth noting that the petal-like intensity structure of the inner ring, which is in the vortex state, rotates in the same direction as the analyzer (counter-clockwise), while the one of the outer ring (in the anti-vortex state) rotates in the opposite direction, as expected [3].

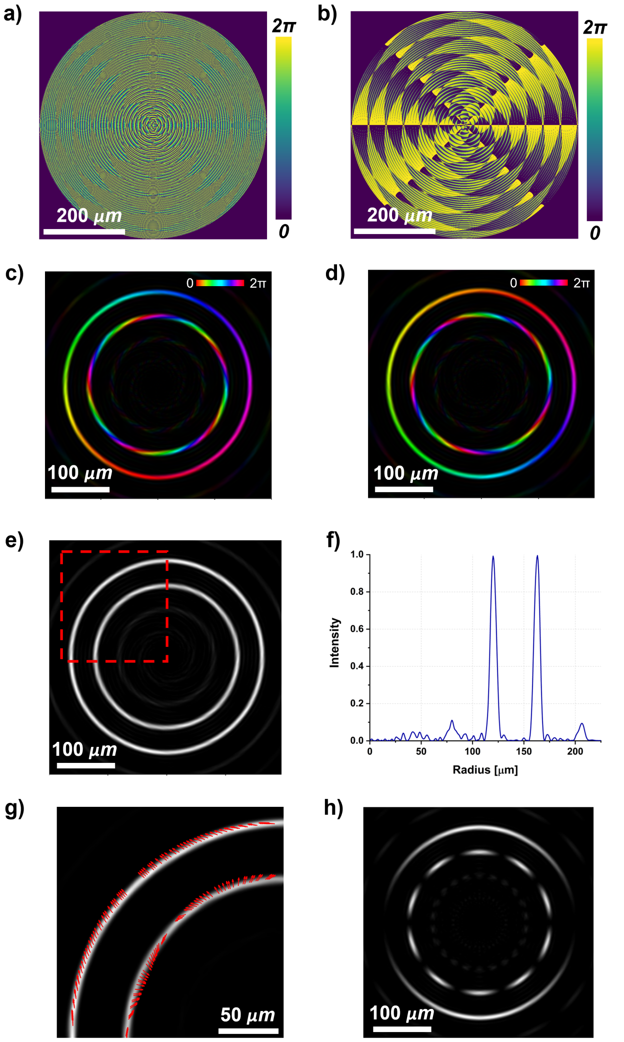

Fig. S4: Simulations of double-ring perfect vector beams (DR-PVBs) generation having rings with different vectorial order, by using the equalized double axicon method. (a) Dynamic phase map δ_x and (b) local nanopillars rotation angle θ required to impart the geometric phase contribution 2θ. Phase and amplitude distributions of the scalar DR-PVs generated under LCP (c) or RCP (d) light in input. Brightness and colours refer to intensity and phase, respectively. Intensity profile (e) and integrated radial profile (f) of the DR-PVB generated with vertically polarized light in input. It can be observed that the dark region between the two rings is larger than the one generated in **3.4 Second method: equalized double axicon** because of the higher value assumed by the parameter α_2 (i.e., 0.520 rather than 0.465). (g) Polarization plot of the upper-left quarter of the beam marked in (e). (h) Petal-like intensity configuration obtained after filtering the beam in (e) with a linear polarizer oriented along the horizontal direction.

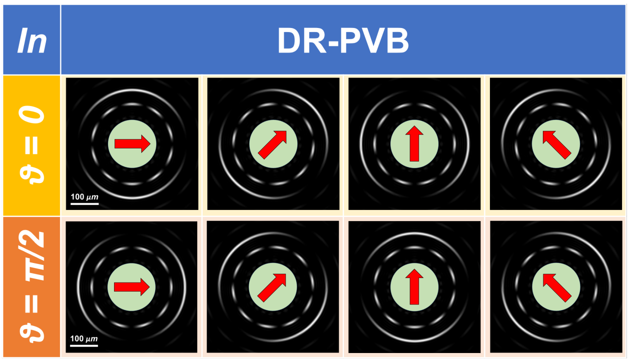

Fig. S5: Characterization of the generated vector states. In the first row (in yellow), we have horizontally polarized light in input, and the figures show the corresponding simulated intensity profiles as a function of the analyzer orientation (depicted by using a red arrow on a green circle). In the second row (in orange) we have vertically polarized light in input. As expected, it is clearly visible that we have opposite rotations of the two petal-like intensity configurations while the analyzer rotates counterclockwise.

References

[1] J. W. Goodman, *Introduction to Fourier optics*. Roberts and Company publishers, 2005.
[2] J.-M. Jin, *The finite element method in electromagnetics*. John Wiley & Sons, 2015.
[3] A. Vogliardi, G. Ruffato, S. Dal Zilio, D. Bonaldo, and F. Romanato, "Dual-functional metalenses for the polarization-controlled generation of focalized vector beams in the telecom infrared," *Scientific Reports*, vol. 13, no. 1, p. 10327, 2023.
[4] C. Rosales-Guzmán and A. Forbes, "How to shape light with spatial light modulators," Society of Photo-Optical Instrumentation Engineers (SPIE), 2017.
